# Myocardial Inflammation as Key Mediator of Heart-brain Interaction After Myocardial Ischemia/Infarction: Mechanistic Exploration of Post-Myocardial Infarction Cognitive Dysfunction

**DOI:** 10.2174/011570159X394212250825051955

**Published:** 2025-09-15

**Authors:** Linhan Wang, Meng Mao, Hailong Bing, Wei Xu, Wangli Tian, Xuan Wang, Zhengyuan Xia, Qinjun Chu

**Affiliations:** 1 Department of Anesthesiology and Perioperative Medicine, Zhengzhou Central Hospital Affiliated to Zhengzhou University, Zhengzhou, China;; 2 Department of Anesthesiology, The University of Hong Kong, Hong Kong, China

**Keywords:** Myocardial infarction, neuroinflammation, cognitive dysfunction, cardiac-brain interaction, blood-brain barrier, inflammatory mediators

## Abstract

Myocardial Infarction (MI) is a severe cardiovascular event, causing not only substantial damage to the heart but also potentially exerting a profound impact on brain function through a complex cardiac-brain interaction mechanism. The pathological process of MI encompasses myocardial cell necrosis, inflammatory cell infiltration, and the release of a substantial amount of inflammatory mediators. Through the bloodstream, these myocardial mediators may traverse the Blood-Brain Barrier (BBB), eliciting a neuroinflammatory response that can lead to cognitive dysfunction. This article proposes a critical research direction: investigating whether MI mediates the effects of myocardial-derived mediators on the permeability of the BBB, as well as the potential consequences of these mediators on cognitive functions. This review is aimed at triggering future research to elucidate the underlying mechanisms governing heart-brain interactions after MI in order to facilitate the development of more effective cognitive protection strategies for patients with MI.

## 1 INTRODUCTION

Cognitive dysfunction—characterized by impairments in memory, executive function, and information processing speed—is increasingly recognized as a debilitating comorbidity in Myocardial Infarction (MI) survivors. Prospective clinical study indicates that about 37% of MI patients exhibit measurable cognitive decline within 6 months after the cardiac event, with deficits persisting for years even in the absence of overt stroke [1]. Notably, hippocampal-dependent memory loss and prefrontal cortex-mediated executive dysfunction dominate the clinical phenotype, mirroring patterns seen in early-stage Alzheimer’s Disease (AD) [2-4]. This overlap raises a critical question: Does MI accelerate neurodegenerative pathways, or does it initiate unique neuroinflammatory cascades? Recent studies have increasingly shown that MI is not solely a local cardiac event; it also induces pathophysiological changes, such as a systemic inflammatory response, oxidative stress, and disorders in neurohumoral regulation, which may profoundly affect distant organs, particularly the brain. This heart-brain interaction has become a focal point of contemporary research.

Classical heart-brain axis models attribute post-MI cognitive deficits to two primary mechanisms: (1) cerebral hypoperfusion due to reduced cardiac output, and (2) silent cerebral emboli from left ventricular thrombi [5]. However, patients with Myocardial Infarction (MI) who exhibit preserved Ejection Fraction (EF) still develop cognitive impairment, as evidenced by clinical findings [6]. Such discrepancies underscore the need to explore non-hemodynamic, non-embolic mechanisms—particularly myocardial inflammation-driven neurotoxicity. This study proposes a novel hypothesis, titled "Myocardial-Neuroinflammatory Coupling Hypothesis," which posits that pro-inflammatory factors (*e.g*., IL-1β, HMGB1) and exosomes (carrying miRNA) released from post-Myocardial Infarction (MI) myocardium may penetrate the Blood-Brain Barrier (BBB) or activate autonomic pathways, triggering microglial activation and neuroinflammation. This hypothesis challenges the traditional paradigm that heart-brain crosstalk is solely driven by ischemia or embolism, providing an inflammation-centric explanatory framework for post-MI cognitive dysfunction.

The post-MI pathophysiological cascade exerts multiorgan impacts through intricate cardio-cerebral interactions involving inflammatory cross-talk, oxidative stress amplification, and neurohumoral dysregulation. Elucidating these mechanisms is critical for developing holistic therapeutic strategies that address both cardiac and neurological sequelae. This review systematically examines: (1) post-MI systemic pathophysiological changes, (2) mechanisms underlying MI-induced cognitive impairment and neuroinflammation, and (3) myocardial inflammation-mediated neuroinflammatory pathways—ultimately refining clinical management and future research directions.

This narrative review synthesized evidence from peer-reviewed articles (2010-2024) retrieved *via* PubMed, Web of Science, and Embase, using keywords: 'myocardial infarction AND cognitive dysfunction,' 'neuroinflammation AND heart-brain axis,' 'exosomes AND blood-brain barrier.' Priority was given to: (1) mechanistic studies in animal models or human cohorts, (2) clinical trials with cognitive endpoints, and (3) high-impact reviews proposing novel paradigms. Grey literature and non-English publications were excluded.

## 2 PATHOPHYSIOLOGY OF MI AND MIRI (MYOCARDIAL ISCHEMIA REPERFUSION INJURY)

**Fig. (1):**
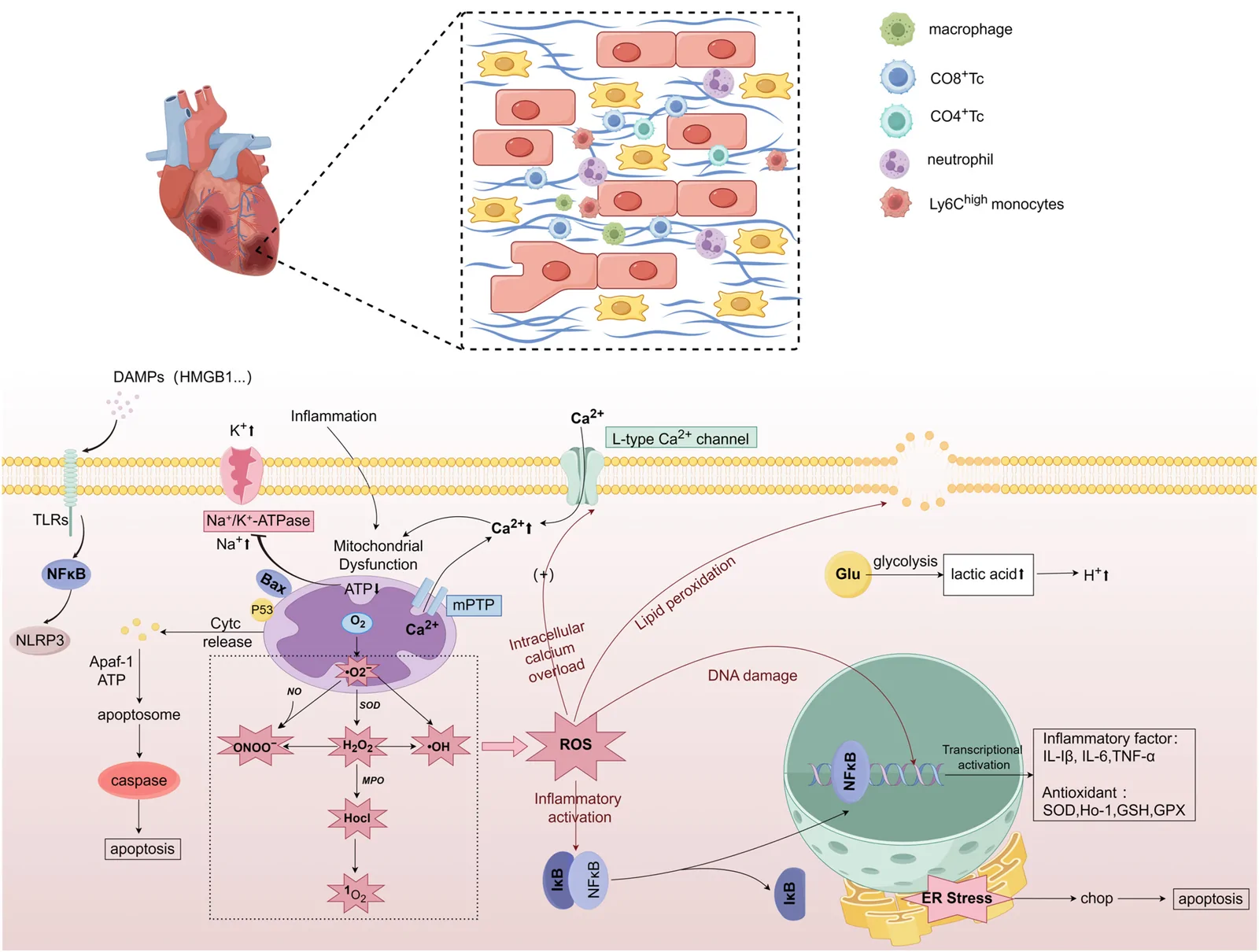
Pathological Alterations of Heart Following Myocardial Infarction. Myocardial cell death following MI results in the release of Damage-Associated Molecular Patterns (DAMPs), which activate the immune system and recruit inflammatory cells, including neutrophils and Ly6c^hi^ monocytes. The infiltrated immune cells, along with damaged mitochondria, produce substantial amounts of Reactive Oxygen Species (ROS), exacerbating oxidative stress and inflammatory responses. The activation of the Nuclear Factor kappa-light-chain-enhancer of activated B cells (NF-κB) pathway promotes the expression of inflammatory factors, such as TNF-α, IL-1β, and IL-6, thereby further amplifying the inflammatory response. Concurrently, energy depletion and ion imbalance lead to myocardial cells undergoing irreversible necrosis. Mitochondrial dysfunction results in the release of cytochrome c, which activates the caspase cascade and triggers programmed cell death. Additionally, intracellular Ca^2+^ overload leads to an accumulation of mitochondrial Ca^2+^, which adversely affects mitochondrial function. This dysfunction exacerbates the energy crisis, leading to reduced ATP production and creating a vicious cycle. Increased anaerobic glycolysis results in the accumulation of lactic acid and protons, which contribute to intracellular acidosis and further impair myocardial cell function. (By Figdraw: TRORS5bebe).

The pathophysiological changes following MI are intricate and multi-layered. Acute myocardial ischemia and necrosis can trigger a cascade of reactions, including oxidative stress, inflammatory responses, metabolic change, neuroinflammation, and apoptosis. These changes not only impact the survival and functional recovery of cardiomyocytes but may also lead to myocardial remodeling and Heart Failure (HF). Furthermore, while reperfusion therapy after MI can restore blood flow, it may also induce Myocardial Ischemia-Reperfusion Injury (MIRI), exacerbating myocardial damage (Fig. **1**).

### 2.1 Oxidative Stress

After MI, restoration of blood flow to the ischemic myocardium is a critical step in order to rescue the heart; however, reperfusion can also trigger myocardial reperfusion injury. In both scenarios, oxidative stress plays a pivotal role, leading to further cellular damage and cardiac dysfunction. Oxidative stress refers to a state in which the balance between Reactive Oxygen Species (ROS) and the body's antioxidant defense system is disrupted, leading to the accumulation of reactive oxygen species and subsequent damage to biological macromolecules, including cells, proteins, lipids, and DNA [7]. In the early stages of MI, ischemia suppresses cellular metabolism, leading to a reduction in ATP production and subsequent cellular dysfunction [7]. The interruption of oxygen supply disrupts the normal function of the mitochondrial electron transport chain, resulting in electron leakage and subsequent reactions with oxygen molecules that produce ROS [8]. These ROS include superoxide anion (O_2_•^−^), hydrogen peroxide (H_2_O_2_), hydroxyl radical (•OH), singlet oxygen (^1^O_2_), and peroxynitrite (ONOO^−^) [9]. Additionally, the activation of enzymes such as xanthine oxidase and NADPH oxidase in ischemic tissue further increases ROS production [10].

A significant amount of ROS is produced during myocardial ischemia/reperfusion injury. These ROS can enter the bloodstream through mechanisms such as the release of cellular contents from injured myocardial cells, leading to damage in brain cells [11]. Furthermore, systemic hypoxia or reperfusion can induce oxidative stress in distant organs, including the brain [12].

ROS can interfere with the homeostasis of intracellular calcium ions, leading to calcium ion overload. This overload activates a variety of calcium-dependent enzymes, including proteases and phospholipases, which further exacerbate cellular damage [13, 14]. ROS can activate the inhibition of Inhibitor of Kappa B (IκB) kinase complex, leading to the degradation of IκB, which releases the IκB on Nuclear Factor kappa-light-chain-enhancer of activated B cells (NF-κB) and facilitates the translocation of NF-κB from the cytoplasm to the nucleus [15]. These inflammatory mediators further amplify the inflammatory response and attract immune cells, such as neutrophils and monocytes, to the site of myocardial injury [16].

### 2.2 Inflammation

Ischemia triggers a cascade of inflammatory responses. DAMPs are endogenous molecules released when cells are damaged or die. These molecules include High Mobility Group Protein B1 (HMGB1), heat shock proteins, ATP, and others. In the context of MI, DAMPs are released into the extracellular environment, activating the innate immune system. By binding to pattern recognition receptors such as Toll-Like Receptors (TLRs), these molecules activate the myeloid differentiation primary response gene 88 (MyD88) and the NF-κB, thereby inducing the production of multiple inflammatory mediators, including interleukin-1 beta (IL-1β) and interleukin-18 (IL-18) [17, 18]. TLRs are primarily located on circulating immune cells and resident cardiac cells, which recognize nucleic acids from bacteria or viruses [17]. Inhibition of TLR2 or TLR4 in animal studies has been shown to reduce the extent of MI [18-23], suggesting that these specific TLRs may serve as potential therapeutic targets for MI.

The NLR family pyrin domain-containing 3 (NLRP3) inflammasome is a multiprotein complex composed of NLRP3, apoptosis-associated speck-like protein, and caspase-1. Its primary function is to NLRP3 inflammasome. Additionally, stress signals such as ROS, potassium ion efflux, mitochondrial dysfunction, lysosomal injury, and extracellular ATP released by injured cells also contribute to NLRP3 activation [24]. The activation of the NLRP3 inflammasome is closely associated with cardiac dysfunction and poor prognosis following MI. Studies have demonstrated that inhibiting the NLRP3 inflammasome can effectively reduce the size of MI [25-29]. Furthermore, NLRP3 inflammasome expression is elevated in cases of MIRI. If intervention occurs at the onset of reperfusion or within one hour thereafter, the size of the MI after 24 hours can be significantly reduced [30].

The complement cascade is activated following Acute Myocardial Infarction (AMI) in response to the release of cardiomyocyte contents, including DAMPs [31]. Complement component C3b plays a crucial role in regulating phagocytes to eliminate foreign and damaged substances. Additionally, complement components C3a, C4a, and C5a exhibit chemotactic activity, attracting a variety of immune cells, including neutrophils, monocytes, macrophages, and mast cells. The complement component C5b-9 is responsible for activating the membrane attack complex, which leads to cell lysis. Numerous experimental animal studies on AMI have demonstrated that genetic or pharmacological inhibition of various components of the complement cascade—such as complement receptor 1 [32], C1 inhibitors [33], mannose-binding lectin [34], and C5 and C5a [35, 36] — can attenuate MIRI.

Neutrophils are the first responders in the inflammatory process. They are attracted to damaged heart muscle tissue through chemokines and release a variety of cytokines and enzymes [37]. These substances are crucial for removing bacteria and cellular debris; however, they can also lead to secondary tissue damage. Study revealed that depletion of neutrophils in mice subjected to MI resulted in worsened heart function, increased fibrosis, and a progressive development of HF. The expression of the phagocytosis receptor myeloid-epithelial-reproductive tyrosine kinase (MerTK), a marker of reparative M2 macrophages, was found to be reduced in the hearts of neutrophil-depleted mice [38]. This suggests that neutrophils play a significant role in post-Myocardial Infarction (MI) cardiac repair by polarizing macrophages towards a reparative phenotype. Additionally, another study demonstrated that neutrophils collected on the first day of MI exhibited high expression of pro-inflammatory markers and were polarized by TLR4, while those collected between the 5^th^ and 7^th^ days post-MI exhibited anti-inflammatory characteristics [39]. This illustrates their dual role in both myocardial damage and repair.

Inflammatory cells, including monocytes and macrophages, migrate to the injured area to eliminate dead cardiomyocytes through phagocytosis and efferocytosis. This process is crucial for preventing further inflammation and promoting tissue regeneration [40]. Macrophages, as pleiotropic cells of the innate immune system, regulate inflammatory responses by releasing a variety of cytokines and chemokines. Over time, the role of macrophages has evolved to support healing and tissue repair. Experimental studies have demonstrated that targeting proinflammatory monocytes or M1 macrophages to inhibit the proinflammatory phase following MI yields cardioprotective effects [41-43]. However, early depletion of monocyte-derived macrophages or inhibition of macrophage migratory function results in inadequate repair after MI and pathological ventricular remodeling [44]. Following MI, MerTK is specifically expressed in reparative M2 macrophages with low levels of lymphocyte antigen 6 complex (Ly6C), contributing to the resolution of inflammation. Conversely, the loss of MerTK in macrophages leads to a marked reduction in their ability to engulf cardiomyocytes, thereby impacting the subsequent myocardial repair process [45]. Additionally, macrophages influence the activity of other immune cells after MI. For instance, M1 macrophages enhance T cell activation by producing cytokines such as IL-1β and IL-23. These cytokines promote the differentiation and proliferation of T cells, particularly the activation of CD8+ T cells, which is associated with a prolonged myocardial recovery time [46]. Additionally, the secretion of IL-23 drives γδ T cells to produce IL-17A, further influencing the immune environment of the myocardium [47].

### 2.3 Metabolic Change

During a cardiac ischemic attack, cardiac metabolism undergoes rapid changes, with hypoxia inhibiting the oxidative metabolism of fatty acids, carbohydrates, ketones, and amino acids while activating anaerobic glycolysis. Upon reperfusion, the influx of oxygen and the removal of ischemic metabolites result in a sudden normalization of intracellular pH, the initiation of oxidative metabolism of various substrates, and ongoing alterations in aerobic glycolysis. The activation of metabolic pathways, including glycolysis [48, 49], ketone oxidation [50], the hexosamine biosynthesis pathway [51], and deacetylation [52], is anticipated to mitigate acute Ischemia-Reperfusion Injury (IRI). These metabolic changes occur throughout the cell, with mitochondria serving as a central focus. Notable alterations include modifications in fatty acid and succinate metabolism, as well as changes in FOF1-ATP synthetase activity, which lead to increased mitochondrial ROS production and subsequent opening of the mitochondrial Permeability Transition Pore (mPTP). Prolonged opening of the mPTP results in mitochondrial swelling and rupture of the outer mitochondrial membrane, ultimately triggering apoptosis and necrosis. This form of cell death is particularly pronounced in cardiomyocytes, which have exceptionally high energy demands [53].

Glycolytic metabolism analysis revealed that the expression of glycolytic-related genes increased on the first day following MI. In contrast, the expression of tricarboxylic acid cycle genes increased on days 3 and 7 after MI, while the expression of genes related to the pentose phosphate pathway also rose on day 7 post-MI. This metabolic reprogramming was observed in monocyte-derived macrophages, but not in resident macrophages [54].

Under ischemic conditions, cardiomyocytes primarily rely on oxidative phosphorylation for energy production. However, ischemia results in a deficiency of oxygen and nutrients, prompting cardiomyocytes to shift to anaerobic glycolysis for energy generation. This transition in cellular metabolism leads to a shift from mitochondrial oxidative phosphorylation to anaerobic glycolysis, resulting in the accumulation of lactic acid and protons within the cell, which subsequently causes a decrease in intracellular pH. This phenomenon is supported by numerous clinical studies that have reported elevated levels of circulating lactate in patients experiencing MI [55, 56]. Furthermore, research indicates that lactic acid is not merely a by-product of energy metabolism; it also functions as a signaling molecule that influences remodeling and adaptive responses in the heart. Specifically, lactic acid has been shown to exacerbate oxidative stress by inhibiting the activity of antioxidant enzymes in cardiac tissue and downregulating the expression of genes associated with lactic acid transport, such as Monocarboxylate Transporter 1 [57]. Additionally, lactic acid plays a role in the onset and progression of AMI through lactate modification. For instance, in the early stages of AMI, H3K18la in monocytes promotes the expression of genes such as Lrg1, Vegf-a, and IL-10, which are crucial for heart repair, and enhance the recovery process of the infarcted myocardium [58]. Conversely, following AMI, the transcription factor Snail1 induces the expression of the TGF-β gene in endothelial cells, thereby initiating endothelial-to-mesenchymal transformation, which negatively impacts cardiac repair [59]. Intracellular proton accumulation activates Na^+^/H^+^ exchangers on the cell membrane, prompting the cell to expel protons while absorbing Na^+^. Concurrently, ATP consumption decreases Na^+^/K^+^ ATPase activity, which exacerbates intracellular Na^+^ overload. Consequently, the Na^+^/Ca^2+^ exchanger operates in reverse mode, attempting to excrete excess Na^+^ from the cell. However, this process leads to an overload of intracellular Ca^2+^ [60].

### 2.4 Apoptosis

During MI, mitochondrial damage can lead to increased permeability of the mitochondrial membrane, resulting in the release of pro-apoptotic factors, such as cytochrome c, from the mitochondria into the cytoplasm. Cytochrome c binds to the apoptotic protease-activating factor and activates the caspase cascade, ultimately leading to apoptosis [61]. Additionally, MI can induce cell surface death receptors, such as Fas receptors, to bind to their ligands and activate caspase-8, which subsequently activates downstream caspase-3 and other effector caspases, ultimately resulting in apoptosis [62]. Heart muscle cells are adversely affected by a deficiency in oxygen and nutrient supply, resulting in disturbances in protein synthesis and folding. This condition facilitates the accumulation of unfolded proteins in the endoplasmic reticulum (ER), thereby triggering the ER stress response. Endoplasmic reticulum stress can induce apoptosis through the activation of pro-apoptotic factors such as C/EBP Homologous Protein (CHOP). The upregulation of CHOP is linked to various cell death pathways, which may further exacerbate dysfunction following a MI [63].

### 2.5 Neuroinflammation after MI

Microglia and astrocytes are essential immunomodulatory cells in the Central Nervous System (CNS). While they typically maintain homeostasis within the nervous system, these cells become aberrantly activated in response to systemic inflammatory events, such as MI [64]. Upon activation, microglia and astrocytes release a variety of inflammatory mediators, including cytokines and chemokines. The excessive release of these mediators not only exacerbates neuroinflammation within the CNS but also contributes to neuronal damage and cognitive dysfunction. The systemic inflammatory response initiated by MI impacts the CNS through the bloodstream. Specifically, proinflammatory cytokines, including tumor necrosis factor-alpha (TNF-α) and interleukin-1β (IL-1β), have been demonstrated to impact endothelial cells directly, thereby enhancing their permeability to solutes. These cytokines induce structural and functional alterations in endothelial cells by activating specific signaling pathways, thereby facilitating the penetration of various substances [65]. This increased permeability allows a greater influx of inflammatory mediators and harmful substances into the brain, intensifying neuroinflammation and neuronal damage. Activated microglia and astrocytes perpetually release inflammatory mediators, establishing a vicious cycle. These mediators not only inflict direct damage on neurons but also disrupt synaptic transmission and neuroplasticity, resulting in synaptic dysfunction. Synaptic dysfunction is a critical mechanism underlying cognitive impairment, as it directly influences the transmission and processing of information. Elevated oxidative stress resulting from MI can lead to the overproduction of free radicals, which attack neuronal cell membranes, proteins, and DNA, ultimately causing apoptosis and necrosis of neuronal cells [66]. The neuronal damage induced by oxidative stress can further aggravate neuroinflammation, creating a self-reinforcing cycle. Persistent neuroinflammation and neuronal damage can lead to synaptic loss and disruption of neural networks, which are closely linked to cognitive dysfunction [67]. Patients may exhibit symptoms such as memory loss, inattention, and executive dysfunction. Over time, these cognitive impairments can significantly impact the quality of life and rehabilitation process of patients. In conclusion, neuroinflammation following MI is a complex phenomenon involving multiple factors and pathways. This process encompasses not only the release of inflammatory mediators and the disruption of the BBB, but also the abnormal activation of immune cells and neuronal damage. These factors are interrelated and collectively contribute to the onset and progression of cognitive dysfunction. Neuroinflammation resulting from MI will be discussed in detail in the subsequent chapters addressing cognitive impairment.

### 2.6 Impact of MI or MIRI on BBB and the Potential Mechanism

The BBB is a complex structure composed of brain microvascular endothelial cells, pericytes, and astrocytes, which serves to protect the brain from harmful substances such as toxins, pathogens, and inflammatory mediators, while permitting the passage of essential nutrients and gases. The integrity of the BBB relies on the tight connections between endothelial cells. Following MI, decreased blood flow and hypoxia induce stress responses in the endothelial cells of the BBB. Oxidative stress disrupts the ion gradient of these endothelial cells, resulting in intracellular calcium overload. This overload activates proteases, such as calcium-dependent proteases, leading to the degradation of tight junction proteins, including occludin and claudins, and thereby compromising the tight junctions between endothelial cells [68, 69]. Furthermore, after MI, hypoxia and the release of inflammatory mediators, such as IL-17A [70] and TNF-α [71], contribute to the reduced expression of these junction proteins, which increases intercellular space and enhances permeability.

Connexin 43 (Cx43) is a key connexin protein that forms gap junctions between cardiomyocytes. Studies have demonstrated that Cx43 exacerbates MIRI by promoting ferroptosis in the context of diabetes. In the early stages of diabetes, the observed reduction in Cx43 expression may contribute to the myocardium's enhanced tolerance to MIRI. Conversely, in the later stages of diabetes, the upregulation of Cx43 leads to increased myocardial damage [72]. HMGB1 is a non-histone nuclear protein that is typically located in the nucleus but is released into the extracellular space during cellular damage or death, becoming a significant DAMP. Systemic inflammation resulting from MI can lead to the widespread release of HMGB1, which subsequently reaches the brain *via* the bloodstream [73, 74]. A study has demonstrated that HMGB1 expression is elevated in the early stages of MI, significantly enhancing the permeability of primary human brain microvascular endothelial cells and the human cerebrovascular endothelial cell line, a widely utilized *in vitro* model of the BBB [75]. In addition to increasing BBB permeability through the activation of inflammatory factors, HMGB1 can also induce the expression of matrix metalloproteinases (MMPs) [76, 77], which degrade extracellular matrix components and tight junction proteins [78]. This further compromises the integrity of the BBB, leading to neuroinflammation and neuronal damage.

## 3 COGNITIVE DYSFUNCTION AFTER MI AND/OR CARDIAC SURGERY

There is growing evidence that MI accelerates cognitive decline. A study involving 4,971 participants found that individuals with a prior history of MI exhibited poorer cognitive function, irrespective of age or education level [79]. Further analysis indicates that the risk of dementia associated with MI is elevated [80, 81]. It has been revealed in a similar study that the likelihood of developing dementia for those with a history of MI is five times greater compared to those without such a history [82].

There is increasing evidence of cognitive decline in patients following heart surgery. Research indicates a temporary decline in cognition—encompassing memory, executive function, and motor speed—shortly after CABG; however, this decline is typically reversible within two weeks [83, 84]. Late cognitive decline after CABG surgery is observed between one and five years post-surgery. Controlled studies have demonstrated that this decline is associated with the use of cardiopulmonary bypass [85, 86], irrespective of the underlying progression of cerebrovascular disease or age-related changes.

Neurological and cognitive dysfunction has been observed in animals following MI, particularly manifested as a loss of motor function in the early stages of the event, along with decreased distance, total distance, and time spent in the open field test centers. Cognitive impairment was noted at 4, 6, 8, and 12 weeks post-MI [87]. In the Morris water maze test, MI rats exhibited a reduced frequency of crossing the platform, a decreased percentage of path length, and less time spent in the target quadrant during the probe test, all of which indicate cognitive impairment. Additionally, in reversal tests, MI rats demonstrated deficits in spatial reverse learning and executive function [88]. The Novel Object Recognition test, which assesses hippocampal-dependent cognitive function, revealed that male mice spent less time exploring novel objects after MI, suggesting hippocampal-dependent cognitive impairment [89].

## 4 MECHANISMS OF HEART-BRAIN INTERACTION AFTER MI/MIRI

### 4.1 Heart-brain Interaction after MI/MIRI

The role of the heart-brain axis involves intricate interactions among the nervous, endocrine, and immune systems. When the heart sustains damage, such as from an MI, the body produces stress hormones that can influence not only the recovery process of the heart but also alter the functions of the brain and its emotional state. The ANS, a component of the human nervous system, regulates various physiological functions that occur without conscious effort, including heart rate, respiration, digestion, and body temperature. This system is divided into two primary branches: the sympathetic nervous system and the parasympathetic nervous system. Research has confirmed that the ANS is essential for the interaction between the heart and the brain [90]. Ischemic events in heart tissue contribute to increased oxidative stress, along with elevated levels of adenosine and other cardiac mediators, which subsequently activate cardiac afferent neurons.

Following an MI, both mechanical and chemical receptors in the heart, including pressure and chemical receptors, become stimulated. Signals are conveyed to the brainstem and hypothalamus, among other regions of the CNS, through the vagus nerve and sympathetic nerve pathways. Cardiac sympathetic nervous system dominance refers to an abnormal increase in sympathetic nervous system activity, resulting in sympathetic nerve influence over the heart that exceeds normal levels, which is characterized by an increased density or number of sympathetic nerves. The onset of cardiac sympathetic nervous system dominance after a MI primarily occurs in the marginal zone, beginning on the third day post-event and lasting for at least three months [91-94]. Excessive sympathetic nerve activation due to MI leads to a significant release of catecholamines, such as adrenaline and norepinephrine. Studies have indicated that sympathetic nerve fibers release Nerve Growth Factor (NGF), which promotes their survival and differentiation [95]. However, prolonged exposure to catecholamines may result in decreased expression of nerve growth factors, leading to sympathetic fiber loss and excessive activation of NLRP3 inflammasome signaling, which subsequently promotes IL-1β production and cardiac adaptive hypertrophy [96]. NGF plays a crucial role in neuroplasticity, and alterations in its expression can affect the survival and function of neurons, potentially resulting in diminished regenerative capacity and impacting cognitive function and emotional stability [97-99]. Consequently, dysregulation of the ANS may lead to difficulties in emotion regulation, manifesting as psychological symptoms such as anxiety and depression. These emotional challenges not only diminish the patient's quality of life but also exacerbate structural brain changes, including accelerated loss of gray matter [100].

Autonomic nervous system activation also stimulates the Hypothalamic-Pituitary-Adrenal (HPA) axis, triggering a cortisol response that correlates with the severity of the lesion and subsequently influences mood and behavior [101]. Research indicates that neurotransmitter activity in the hypothalamus undergoes significant alterations following MI. For instance, neurons originating from the hypothalamic arcuate nucleus (ARC) may exhibit reduced excitatory transmission after Myocardial Infarction (MI). Furthermore, the excitatory transmission of paraventricular nucleus-oxytocin (PVN-OXT) neurons from the hypothalamus to parasympathetic dorsal motor nucleus of the vagus (DMNX) neurons is inhibited, suggesting that MI may compromise hypothalamus-related neural networks. This alteration could impact cardiac function by modulating the activity of parasympathetic nerves, particularly in regulating heart rate and blood pressure. Notably, neurons in the prefrontal cortex and hypothalamus show increased sensitivity to apoptosis during the initial 2 to 3 weeks following MI, while the hippocampus, although relatively protected, may still be indirectly affected. The rise in apoptosis suggests a failure of neuroprotective mechanisms, thereby exacerbating damage and dysfunction within the limbic system [102].

The hippocampus is a brain region closely associated with memory and learning. Studies have demonstrated that hippocampal dendritic length and spinous process density are reduced following MI. These alterations are associated with a decreased expression of neuronal cystic fibrosis transmembrane conductance regulators, which may affect hippocampal function and structure [103]. In some patients with MI, alterations in cortical thickness have been observed. For instance, one study indicated that patients with MI exhibited increased cortical thickness but decreased cortical surface area and total white matter, potentially affecting their cognitive function and emotional state [104].

Additionally, immune responses can influence the interaction between the heart and brain through the heart-brain axis, contributing to the development of chronic diseases. Utilizing a Data-Independent Acquisition (DIA) mode proteomics approach, researchers have discovered that MI may induce significant alterations in protein expression within brain and heart tissues, potentially linked to cellular damage and inflammatory responses [105]. Studies indicate that a rapid inflammatory response following acute MI may hinder neurogenesis, the process of forming new neurons, thereby impacting neuroplasticity and the brain's recovery capacity. The specific neuroinflammatory mechanisms have been thoroughly discussed in the preceding sections and will not be reiterated here [106].

Emerging evidence reveals that the timing of MI onset critically shapes inflammatory responses through circadian mechanisms. In murine models, sleep-phase MI (induced during the light period) preferentially activates inflammatory genes, while wake-phase MI triggers metabolic pathways. This temporal divergence correlates with neutrophil infiltration patterns and 7-day survival rates, indicating that cardiac repair is circadian-gated [107].

Crucially, MI dynamically reprograms brain-immune crosstalk *via* monocyte trafficking. Within hours post-MI, circulating monocytes are recruited to the Lateral Posterior thalamic Nucleus (LPN) through choroid plexus signaling. These monocytes produce TNF-α to activate TNFRSF1A-expressing glutamatergic neurons, thereby amplifying the pressure of Slow-Wave Sleep (SWS). Enhanced SWS suppresses cardiac sympathetic outflow *via* β-adrenergic receptor signaling on macrophages, thereby reducing myocardial inflammation and improving survival [108]. This monocyte-sleep-cardiac axis represents a novel self-protective circuit: heart injury - monocyte brain recruitment - sleep induction - sympathetic suppression - cardiac healing.

### 4.2 Current Understanding of the Mechanism of Cognitive Dysfunction

Relevant studies have elucidated the underlying mechanisms of brain pathological changes following MI. Factors such as oxidative stress, neuroinflammation, glial activation, dendritic spine loss, neurohormonal activation, and brain cell death are considered to contribute to cognitive dysfunction in patients who have experienced MI.

A significant decrease in cerebral blood flow is a common occurrence following MI. A study demonstrated that cerebral blood flow decreased markedly after MI, and this reduction remained significant for the subsequent 30 days [109]. Cerebral hypoperfusion is one of the predominant causes of cognitive impairment post-MI, and animal models have indicated diminished cardiac output and cerebral blood flow in mice subjected to ligation of the left anterior descending coronary artery [109, 110]. Furthermore, brain blood flow and glucose metabolism are notably reduced in patients with Alzheimer's Disease (AD). Early asymptomatic AD is characterized by cognitive decline resulting from inadequate cerebral perfusion, and enhancing cerebral blood flow has been shown to alleviate Alzheimer's symptoms [111, 112]. This evidence further supports a robust association between cerebral hypoperfusion and cognitive decline.

Hypoperfusion can result in structural changes in the brain. Patients with congestive HF are particularly susceptible to brain parenchymal loss, especially in gray matter, due to low cerebral blood flow [113, 114]. This susceptibility arises because the higher metabolic requirements of gray matter necessitate more than two-thirds of cerebral blood flow [115]. Research indicates that the volume of gray matter in the brain is closely linked to cognitive ability; thus, a reduction in gray matter volume is associated with damage to various cortical regions involved in executive cognitive function [116]. Magnetic Resonance Imaging (MRI) studies have demonstrated a strong correlation between cerebral white matter lesions, brain atrophy in AD patients, and cerebral hypoperfusion [117]. Furthermore, interventions aimed at improving heart function, such as heart transplantation or resynchronization therapy, have been shown to enhance cognitive function [118]. Consequently, cardiac dysfunction can lead to hypoperfusion, ultimately resulting in cognitive impairment.

Chronic cerebral hypoperfusion can result in brain cell hypoxia, disturbances in cellular metabolism, mitochondrial dysfunction, and the production of excessive ROS. These reactive species, including superoxide and nitric oxide, activate MMPs such as MMP-2 and MMP-9, which can degrade extracellular matrix components and tight junction proteins, including occludin and claudins. This degradation leads to oxidative damage to cellular molecules, promotes inflammatory cytoskeletal reorganization, and compromises the integrity of the BBB [119]. The disruption of the BBB is a significant pathogenic mechanism underlying cognitive impairment. Following BBB rupture, oxidative stress and inflammation can be exacerbated within the brain, triggering further oxidative stress, microglial overactivation, and neuronal cell death. This sequence of events results in the loss of dendritic spines [120], which directly correlates with cognitive decline.

## 5 MYOCARDIAL INFLAMMATION-NEUROINFL-AMMATION REINFORCES TO IMPACT ON COGNITIVE DYSFUNCTION AND MI

### 5.1 Neuroinflammation as a Key Mechanism of Cognitive Dysfunction

**Fig. (2):**
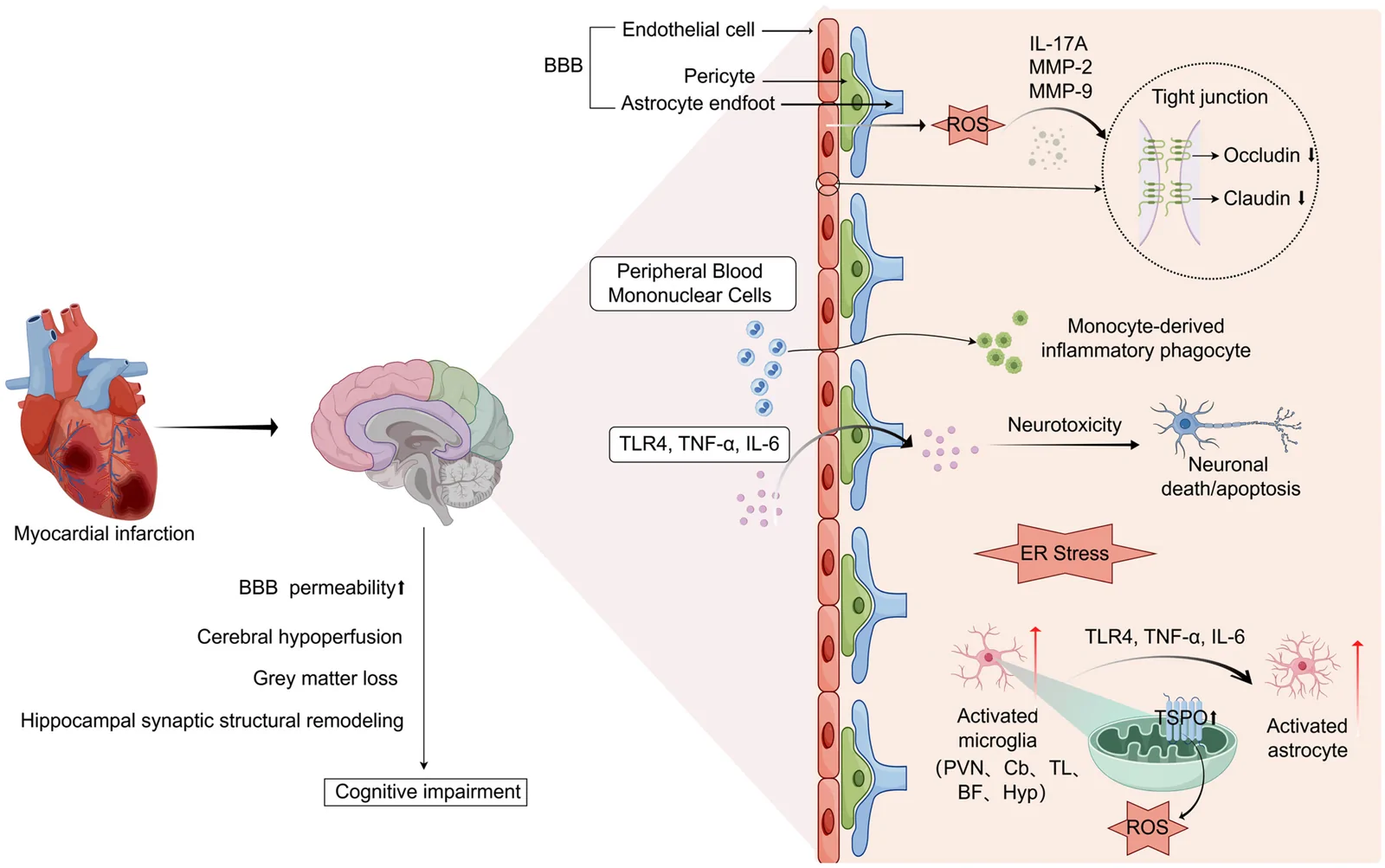
Neuroinflammation as a Mediator of Post-Myocardial Infarction Cognitive Impairment. MI results in reduced cerebral perfusion, loss of gray matter, and decreased dendrite length and spine density in the hippocampus. Circulating inflammatory factors, such as TNF-α and IL-1β, can directly affect cerebral vascular endothelial cells, activating oxidative stress and leading to a reduction in the expression of intercellular tight junction proteins, including claudin-5 and occludin. The increased permeability of the Blood-Brain Barrier (BBB) facilitates the entry of additional inflammatory factors, such as TLR4, TNF-α, and IL-6, as well as peripheral immune cells, including neutrophils, monocytes, and T cells, into the brain parenchyma. The infiltration of these peripheral inflammatory factors and immune cells activates microglia, transitioning them from a resting to an activated state. Activated microglia release elevated levels of pro-inflammatory cytokines, including TLR4, TNF-α, and IL-6, as well as neurotoxic substances such as ROS. This activation further stimulates astrocytes and exacerbates neuroinflammation. The resulting neuroinflammation and infiltration of immune cells contribute to increased oxidative stress, ultimately impairing neuronal and synaptic function, which may lead to cognitive deficits. (By Figdraw: YUYUObe587). **Abbreviations**: (BBB, blood-brain barrier; MMP, Matrix Metalloproteinase; PVN, Paraventricular Nucleus; Cb, Cerebellum; TL, Temporal Lobe; BF, Basal Forebrain; Hyp, Hypothalamus; ER, Endoplasmic Reticulum; ROS, Reactive Oxygen Species; TLR4, Toll-Like Receptor 4; TNF-α, Tumor Necrosis Factor-Alpha; IL-6, Interleukin-6; TSPO, translocator protein).

Cerebral hypoperfusion resulting from decreased cardiac function can lead to neuroinflammation. Following a heart attack, the body initiates a systemic inflammatory response due to damage to heart tissue and cell death, which results in the release of inflammatory factors (Fig. **2**). These factors are not confined to the heart; they also enter the circulatory system, potentially affecting other organs, including the brain. Within the brain, these inflammatory factors can induce neuroinflammation, leading to a decline in nerve cell function and ultimately, cell death [121]. A study has indicated that the abundance of monocytes in the brain increases after MI, which correlates with the proliferation of macrophages and microglia surrounding the brain's resident blood vessels. Inhibition of monocyte recruitment has been shown to preserve white matter integrity and cognitive function [122], thereby linking monocytes to neurodegeneration following MI. Additionally, increased BBB permeability facilitates the adhesion and migration of monocytes from the periphery to the CNS, leading to their eventual differentiation into microglia and exacerbating neuroinflammation [123]. Notably, spatially resolved mechanisms reveal dual functionalities of these infiltrating monocytes: In perivascular regions, monocytes differentiate into macrophages that amplify microglial activation and drive white matter injury *via* CCL2-dependent oligodendrocyte dysfunction [122]. In the LPN, monocytes release TNF-α to activate glutamatergic neurons, enhancing SWS. This SWS augmentation suppresses cardiac sympathetic hyperactivity, thereby limiting myocardial inflammation and improving survival [108]. This anatomic divergence necessitates targeted therapeutic strategies: interventions preserving thalamic monocyte-mediated SWS while inhibiting perivascular monocyte neurotoxicity.

Research conducted in animal models has demonstrated that microglial phenotypes change in various brain regions after MI. In rats, the morphology of microglia underwent significant changes following MI in the rostral ventrolateral medulla (RVLM), Nucleus Tractus Solitarius (NTS), and periaqueductal gray matter (PAG), all of which play crucial regulatory roles in cardiovascular function [124]. Furthermore, an increase in the number of activated microglia in the hypothalamic paraventricular nucleus (PVN) was observed between 2 and 16 weeks post-MI [125, 126]. Elevated levels of proinflammatory cytokines in the PVN activate the hypothalamic-pituitary-adrenal axis, enhance sympathetic nervous system activity, and contribute to acute proinflammatory responses following MI [127]. Additionally, studies utilizing positron emission tomography have imaged early and late microglial activation after MI in humans. The findings revealed enhanced signaling of the translocator protein (TSPO) in the cerebellum, temporal lobe, frontal basal cortex, and hypothalamus, indicating an increase in activated microglia in these areas [128]. Furthermore, microglial activation in the PVN, RVLM, PAG, and NTS regions was effectively reduced by at least 50% following intravenous administration of minocycline one week prior to MI. Concurrently, neuron activation in the PVN was also significantly reduced by 50%, while neuron activation in the PAG, RVLM, and NTS was nearly eliminated [124]. These results suggest that MI may trigger microglial activation, which in turn promotes increased neuronal activity.

Synaptic impairment is closely associated with cognitive decline and memory loss. Following myocardial infarction, neurons in the central nervous system exhibit substantial structural remodeling [129]. Studies have demonstrated that MI induces morphological alterations in hippocampal neurons, including reduced dendritic length and decreased spine density, which may contribute to neuronal dysfunction and impaired signal transmission efficiency [103, 130].

Notably, these structural perturbations coincide with systemic inflammatory cascades triggered by cardiac dysfunction. In the HF mouse model, the expression of inflammatory genes, such as TLR4, TNF-α, and IL-6, was significantly upregulated in both the cortex and hippocampus [87]. These inflammatory factors can infiltrate the CNS through a compromised BBB. Following MI, the levels of proinflammatory cytokines in the brain increased, with a notable rise in brain TNF-α expression in microglia observed in a mouse study conducted 6-8 weeks post-myocardial ischemia [131]. TNF-α serves as a principal regulator of the brain's proinflammatory response and can exacerbate neurotoxicity by enhancing neuronal glutamate, leading to cell damage and death [132]. Research indicates that the ASK1-p38-TNF-α pathway is implicated in the disruption of tight junctions following chronic cerebral hypoperfusion [133]. Cerebral hypoperfusion also influences IL-1β levels in the CNS, which plays a critical role in regulating the inflammatory cascade [134]. In the ischemic brain, TNF-α and IL-1β stimulate IL-6 production, with elevated serum and cerebrospinal fluid levels of IL-6 being linked to cognitive dysfunction [135]. Cytokines that are released during the inflammatory response have the potential to trigger cognitive impairment. For instance, IL-17A is elevated after MI and contributes to the disruption of the nuclear BBB in MI rat models [70]. In addition, studies have found that the level of IL-17A in the cerebrospinal fluid of rats with MI is increased in parallel to the increased level of IL-17A in plasma, leading to neuroinflammation in rats with MI [136]. Studies have shown that deletion of IL-17A can improve long-term cognitive impairment caused by anesthetic drugs in neonatal mice, indicating the importance of IL-17A in impaired cognitive function. This suggests that IL-17A may induce long-term cognitive impairment by promoting inflammatory pathways after MI [137]. Additionally, inflammatory mediators may impact neuronal function by disrupting synaptic transmission and the relay of nerve signals. Such dysfunction can result in cognitive and motor impairment, particularly in elderly individuals.

CX3CR1 (C-X3-C chemokine receptor 1) plays a significant role in cognitive function and memory formation. Studies have demonstrated that the CX3CR1 signaling pathway is closely related to neuritis, synaptic pruning, and neural plasticity, all of which directly influence cognitive abilities and memory formation in the brain [138]. Silencing the CX3CR1 gene in microglia has been shown to reduce cytokine release effectively, mitigate white matter damage, and enhance cognitive function [139]. Additionally, studies have demonstrated that leptin can amplify the expression of proinflammatory cytokines in BV2 microglia under hypoxic conditions [140]. Studies have shown that leptin levels increase significantly within 24 hours following AMI, peaking on the second day of hospitalization, often exceeding twice the baseline level observed at admission [141]. This rapid increase in leptin may be closely associated with the inflammatory response and myocardial damage. Furthermore, leptin secretion correlates with the levels of multiple proinflammatory cytokines, particularly during the event of MI [142]. Du *et al*. found that the loss of leptin receptors promotes the expression of anti-inflammatory cytokines in white matter and activates M2-type microglia by inhibiting the activation and pro-inflammatory response of glial cells. This mechanism protects mice from cognitive dysfunction induced by cerebral hypoperfusion and white matter injury [143].

### 5.2 Extracellular Vesicles (EVs) and MicroRNAs (miRNAs)

**Fig. (3):**
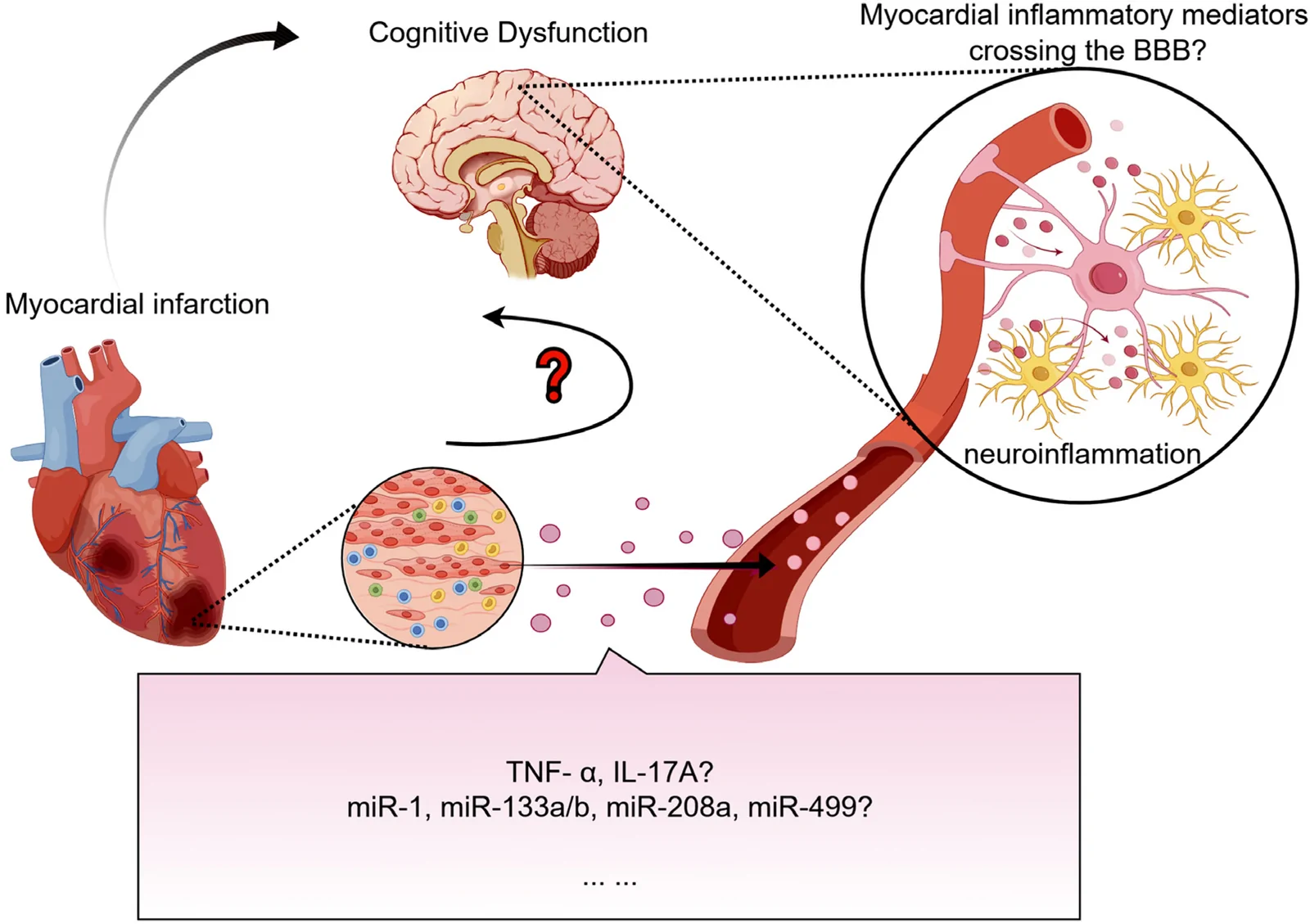
After MI, myocardial-derived factors are likely to serve as potential mediators for triggering post-infarction cognitive impairment. Injured cardiomyocytes following MI release a range of inflammatory mediators into the bloodstream. A pertinent question arises: will these myocardial-derived inflammatory mediators cross the BBB and reach the brain to contribute to neuroinflammation after MI? This topic awaits further exploration. In this context, the study leads one to speculate that certain mediators released from damaged myocardial tissue may traverse the BBB and enter the brain. These mediators include TNF-α, IL-17A, and various microRNAs such as miR-1, miR-133A/B, miR-208A, and miR-499 [157, 158]. (By Figdraw: TAWAU444da).

Following an MI, cardiac cells release a series of specific mediators that enter the brain *via* the bloodstream, subsequently affecting neural activity and function, which can lead to cognitive decline. These mediators include various inflammatory factors and neurotransmitters. Under normal physiological conditions, the efficiency of these mediators crossing the BBB is relatively low. However, during systemic inflammation induced by MI, the integrity of the BBB may be compromised, resulting in increased permeability to these mediators. Furthermore, mediators released by damaged cardiac cells may also traverse the BBB more effectively through transcellular pathways (Fig. **3**).

Extracellular Vesicles (EVs) are recognized as significant mediators of heart-brain interactions. These small vesicle-like structures are released by cells into the extracellular environment and are widely present in body fluids and tissues. EVs transport lipids, metabolites, proteins, ribonucleic acid (RNA), and deoxyribonucleic acid (DNA) from donor cells to distant recipient cells, facilitating intercellular signaling and playing a crucial role in intercellular communication [144]. Various studies have indicated that elevated levels of Extracellular Vesicles (EVs) following MI may serve as a predictor for the future risk of cardiovascular events in patients with coronary artery disease [145, 146]. An increase in cardiomyocyte- and endothelial cell-derived EVs has been observed in the circulation of patients with ST-Elevation MI (STEMI) as well as in mice with MI [147]. Research has demonstrated that EVs can traverse the BBB, circulate from the brain to the peripheral blood, and move from the peripheral blood to the brain [148, 149]. EVs released from the heart during pathological states may impact multiple organs, including the brain. Furthermore, peripheral circulation-derived exosomes can induce the activation of microglia and astrocytes, leading to the expression of inflammatory cytokines in the brain [150]. For instance, serum exosomes rich in miR-15a, miR-15b, miR-21, and miR-155 can traverse the BBB, leading to the induction of small glial hyperplasia. Consequently, levels of miR-155 and proinflammatory cytokines, such as TNF-α and IL-6, are elevated [151]. This suggests an enhanced capacity of extracellular vesicles (EVs) to traverse the BBB during inflammatory conditions. This transport may occur through adsorption-mediated transcytosis. These findings suggest a potential regulatory role of EVs in the function of both neurons and microglia. Studies have demonstrated that circulating Extracellular Vesicles (EVs) containing HMGB1 isolated from patients with Traumatic Brain Injury (TBI) are sufficient to induce endothelial dysfunction and contribute to secondary brain damage in mice [152]. Therefore, extracellular vesicles containing HMGB1 may facilitate interactions between the heart and brain. Circulating inflammatory exosomes can induce neuroinflammation even in the absence of systemic inflammation. Consequently, EVs released from cardiomyocytes following MI are likely to penetrate the BBB, transporting inflammatory factors into the brain. This process may modulate neuroinflammation and cognitive function, potentially leading to anxiety and depression in MI patients, along with various related symptoms, including cognitive deficits.

MicroRNAs (miRNAs) are a class of non-coding RNA molecules approximately 22 nucleotides in length that promote mRNA degradation and inhibit gene expression by binding to the 3' untranslated region (3' UTR) of target mRNAs through incomplete base pairing [153]. MiRNAs have potential as biomarkers, exhibiting temporal and spatial variations in response to different pathological conditions. Unlike intracellular miRNAs, circulating miRNAs demonstrate remarkable stability and resistance to degradation by endogenous ribonuclease activity [154]. Most circulating miRNAs are derived from blood cells, while others originate from various tissues, including the heart, lung, liver, and kidney [155, 156]. Notably, miR-1, miR-133a/b, miR-208a/b, and miR-499 are upregulated in serum following AMI [157, 158]. Among these, miR-1, miR-208, and miR-499 are present in plasma and are carried by exosomes [159]. These miRNAs are highly expressed in the myocardium and are primarily involved in the development, differentiation, and functional maintenance of cardiomyocytes [160]. Although the expression of these miRNAs is specific to the myocardium, some studies suggest their involvement in regulating brain function, indicating a potential mechanism by which MI may affect the brain. For instance, miR-1 is implicated in hypoxia-induced neuronal damage, mediating neuronal apoptotic damage through the intrinsic Bax-mitochondria-caspase protease pathway [161]. Furthermore, inhibition of miR-1 expression has been shown to reduce infarct volume in a rat model of transient Middle Cerebral Artery Occlusion (MCAO) [162]. Additionally, miR-1 may regulate synapse generation, learning, and memory by modulating Brain-Derived Neurotrophic Factor (BDNF) [163]. Specific overexpression of cardiac miR-1 can increase the levels of miR-1 in the hippocampus of the brain, leading to the downregulation of BDNF and subsequent cognitive impairment in mice [164]. The knockdown of hippocampal microRNA-1 has been shown to ameliorate the long-term potentiation damage induced by MI [165]. Additionally, studies indicate that miRNAs can traverse the BBB *via* exosomes [166]. Consequently, it is plausible that exosomes released from the myocardium following MI may enter the brain through the circulatory system, exacerbating cognitive impairment. These findings offer new insights into the effects of MI on brain function and may identify potential targets for future therapeutic strategies.

## 6 FUTURE RESEARCH

There is a growing appreciation for the importance of systemic and circadian processes in modulating various medical conditions [167]. Raised levels of circulating and/or exosomal pro-inflammatory cytokines increase gut permeability and drive gut dysbiosis, with significant consequences mediated by increase LPS and decreased butyrate, respectively [168]. Butyrate is a significant positive modulator of MI recovery and regeneration [169], with wider effects that include the optimization of mitochondrial function [170]. LPS, like pro-inflammatory cytokines, decreases pineal melatonin production [171], thereby allowing inflammatory processes to alter the interactions between the gut and the pineal gland. Melatonin affords significant protection against and recovery from MI [172], as well as melatonin and butyrate having significant impacts on cognition [173, 174]. This would indicate that such systemic-circadian interactions may be significant aspects of MI and how it interfaces with cognition. Interestingly, both melatonin and melatonin-induced oxytocin can activate the vagal nerve, thereby increasing vagal acetylcholine (ACh) that activates acetylcholine receptors [175], including the α7 nicotinic acetylcholine receptor (α7nAChR), with α7nAChR activation being an important suppressor of MI pathophysiological changes [176]. The α7nAChR is also an important modulator of cognition [177]. As pineal melatonin increases the α7nAChR over the circadian rhythm [178], the inflammatory cytokine/LPS suppression of pineal melatonin will suppress the capacity of melatonin to have direct effects in cardiomyocytes, as well as suppress the vagal-ACh-α7nAChR pathway. Melatonin and butyrate also inhibit the nuclear translocation of the Glucocorticoid Receptor (GR), thereby preventing the detrimental effects of GR activation on MI [179] and cognition [180], indicating a role for alterations in melatonin interactions with cortisol at the GR in the course of night-time dampening and resetting for the coming day in MI pathophysiology and its interactions with cognition [181]. Building on the systemic-circadian interactions discussed, we hypothesize a dynamic myocardial inflammation-monocyte-sleep-melatonin-cognition cascade: Myocardial inflammation post-MI elevates circulating DAMPs and cytokines, which compromise BBB integrity. Monocyte brain influx through the disrupted BBB occurs in two distinct compartments: Perivascular monocytes drive neuroinflammation and white matter injury, LPN releases TNF-α to enhance SWS [108]. SWS augmentation may stimulate pineal melatonin secretion through TNF-α activation. Critical unknowns for future research: Does thalamic monocyte-derived TNF-α directly increase melatonin synthesis? Can timed melatonin supplementation amplify SWS-driven cardiocerebral protection while suppressing perivascular monocyte neurotoxicity? Future research clarifying the role of such circadian-systemic interactions should better clarify the complexity of processes involved in MI modulation of cognition.

Emerging evidence positions Fibroblast Growth Factor 21 (FGF21) as a crucial mitokine and adipokine with significant potential to favorably modulate both post-Myocardial Infarction (MI) cardiac pathology and associated cognitive dysfunction. FGF21 exerts potent cardioprotective effects after MI/ischemia-reperfusion injury by attenuating myocardial inflammation and stabilizing mitochondrial integrity [182, 183]. Critically, FGF21 levels are independently associated with acute MI severity and correlate positively with inflammatory markers (IL-6, TNF-α) and coronary stenosis [184]. Therapeutic administration of FGF21 robustly improves cardiac function and reduces inflammation after Myocardial Infarction (MI) in experimental models [183-185].

Beyond direct cardiac benefits, FGF21 effectively crosses the BBB, attributable to its low heparin affinity [186], enabling neuroprotective actions in diverse CNS pathologies, including traumatic brain injury [187], ischemic stroke [188], and depression [189]. Recombinant human FGF21 (rhFGF21) demonstrates significant neuroprotection by modulating microglial activation, shifting polarization from pro-inflammatory (M1) to anti-inflammatory/reparative (M2) phenotypes [190]. Consequently, rhFGF21 treatment ameliorates white matter injury, promotes remyelination, and improves cognitive outcomes in hypoxic-ischemic models [190]. Furthermore, FGF21 release is modulated by mitochondrial stress and protective agents, such as melatonin [191], suggesting interconnected pathways relevant to post-myocardial infarction (MI) brain injury.

Thus, we propose the compelling hypothesis that FGF21, acting as a key mitokine, may favorably modulate post-MI cognitive dysfunction through a dual mechanism: (1) indirectly by attenuating the primary myocardial inflammatory insult and systemic inflammatory cascade, and (2) directly by penetrating the BBB to suppress neuroinflammation, promote microglial M2 polarization, and protect white matter integrity in cognition-critical regions. Future research should prioritize elucidating FGF21's kinetics within the post-MI heart-brain axis, evaluating its direct cognitive benefits, exploring synergy with circadian modulators, and developing enhanced delivery systems. This hypothesis warrants in-depth study to evaluate FGF21-based therapies that protect both the heart and mind after myocardial infarction.

## CONCLUSION

The interplay between myocardial inflammation and post-Myocardial Infarction (MI) cognitive dysfunction represents a critical and emerging area of research that bridges cardiology and neurology. This review has highlighted the multifaceted mechanisms through which myocardial inflammation orchestrates a cascade of events leading to cognitive decline following MI. The inflammatory response initiated in the heart can propagate systemically, crossing the BBB and affecting the brain *via* various pathways, including the release of pro-inflammatory cytokines, microRNAs, and other signaling molecules through exosomes. These mediators can induce neuroinflammation, disrupt neurovascular coupling, and impair synaptic plasticity, ultimately contributing to cognitive impairment.

A critical insight emerging from this synthesis is the bidirectional nature of heart-brain interactions. For instance, systemic inflammation following MI compromises BBB integrity through oxidative stress-mediated degradation of tight junction proteins (*e.g*., occludin, claudins) and activation of MMPs. This breach allows inflammatory mediators, such as HMGB1 and cytokines, to infiltrate the brain, activating microglia and astrocytes. Activated glial cells perpetuate neuroinflammation by releasing additional cytokines and ROS, which impair synaptic plasticity and neuronal survival—particularly in regions critical for cognition, such as the hippocampus and prefrontal cortex. Simultaneously, post-MI ANS dysregulation, characterized by sympathetic overactivation and vagal suppression, heightens systemic inflammation and oxidative stress, further aggravating both cardiac remodeling and cerebral hypoperfusion. The latter reduces cerebral blood flow, exacerbating metabolic stress in neurons and amplifying neurovascular uncoupling, a phenomenon increasingly linked to cognitive deficits in conditions like Alzheimer’s disease.

The role of molecular mediators, particularly miRNAs and EVs, adds another layer of complexity to this interplay. Cardiac-specific miRNAs (*e.g*., miR-1, miR-208, miR-499), released into circulation *via* EVs after MI, can traverse the compromised BBB and directly influence neuronal function. For example, miR-1 has been implicated in hippocampal synaptic dysfunction by downregulating BDNF, a key modulator of neuroplasticity and memory. Similarly, HMGB1-laden EVs from injured cardiomyocytes may activate microglial TLR4/NF-κB signaling in the brain, amplifying neuroinflammatory cascades. These findings highlight the potential for miRNA- and EV-based biomarkers to predict cognitive outcomes after Myocardial Infarction (MI) and for targeted therapies to disrupt pathological heart-brain signaling.

Current therapeutic strategies for MI—such as reperfusion therapies and anti-inflammatory agents—often overlook their neurological consequences. However, interventions targeting myocardial inflammation (*e.g*., NLRP3 inflammasome inhibitors, complement cascade blockers) may confer dual cardioprotective and neuroprotective benefits. For instance, inhibiting the NLRP3 inflammasome not only reduces infarct size but may also attenuate neuroinflammation by limiting the production of IL-1β and IL-18. Similarly, modulating ANS activity through vagal nerve stimulation or β-blockers could mitigate sympathetic overdrive, thereby reducing systemic inflammation and cerebral oxidative stress. Emerging therapies, such as monoclonal antibodies against pro-inflammatory cytokines (*e.g*., IL-17A) or miRNA antagonists (*e.g*., anti-miR-1), hold promise for preserving BBB integrity and synaptic function in at-risk patients.

Future research must also address several unanswered questions. First, the role of non-neuronal cells, such as pericytes and astrocytes, in mediating BBB disruption and neuroinflammation requires deeper exploration. Second, the impact of comorbidities—such as diabetes, hypertension, and aging—on heart-brain crosstalk remains underexplored. For example, diabetic patients exhibit exacerbated MIRI due to enhanced Cx43-mediated ferroptosis, which may synergize with neuroinflammatory pathways to accelerate cognitive decline. Additionally, circadian and systemic interactions, such as melatonin suppression due to inflammation-driven gut dysbiosis, warrant attention. Melatonin and butyrate, both modulated by gut microbiota, have shown cardioprotective and neuroprotective effects by optimizing mitochondrial function and suppressing glucocorticoid receptor activation. Their circadian regulation may influence MI outcomes and cognitive recovery, suggesting novel chronotherapeutic approaches.

In summary, the nexus between myocardial inflammation and cognitive decline post-MI is a paradigm of systemic disease with far-reaching neurological consequences. Deciphering this relationship demands a concerted effort to bridge molecular insights with clinical innovation, ensuring that therapies protect not only the heart but also the mind.

## LIST OF ABBREVIATIONS

AD: Alzheimer's Disease
ANS: Autonomic Nervous System
BBB: Blood-Brain Barrier
BDNF: Brain-Derived Neurotrophic Factor
CABG: Coronary Artery Bypass Grafting
CHOP: C/EBP Homologous Protein
CNS: Central Nervous System
CX3CR1: C-X3-C Chemokine Receptor 1
Cx43: Connexin 43
DAMPs: Damage-associated Molecular Patterns
ER: Endoplasmic Reticulum
FGF21: Fibroblast Growth Factor 21
GR: Glucocorticoid Receptor
HF: Heart Failure
HMGB1: High Mobility Group Protein B1
IL: Interleukin
IκB: Inhibition of Inhibitor of Kappa B
LPN: Lateral Posterior Thalamic Nucleus
Ly6C: Lymphocyte Antigen 6 Complex
MerTK: Myeloid-epithelial-reproductive Tyrosine Kinase
MI: Myocardial Infarction
MIRI: Myocardial Ischemia-reperfusion Injury
MMPs: Matrix Metalloproteinases
MnSOD: Manganese Superoxide Dismutase
NF-κB: Nuclear Factor kappa-light-chain-enhancer of Activated B Cells
NLRP3: NLR Family Pyrin Domain-containing 3
NTS: Nucleus Tractus Solitarius
PAG: Periaqueductal Gray Matter
PVN: Paraventricular Nucleus
ROS: Reactive Oxygen Species
RVLM: Rostral Ventrolateral Medulla
SWS: Slow-wave Sleep
TLRs: Toll-like Receptors
TNF-α: Tumor Necrosis Factor Alpha
TSPO: Translocator Protein
α7nAChR: α-7 Nicotinic Acetylcholine Receptor
